# Health Benefits of Montmorency Tart Cherry Juice Supplementation in Adults with Mild to Moderate Ulcerative Colitis: A Protocol for a Placebo Randomized Controlled Trial

**DOI:** 10.3390/mps6050076

**Published:** 2023-08-27

**Authors:** Jonathan Sinclair, Stephanie Dillon, Robert Allan, Johanne Brooks-Warburton, Terun Desai, Charlotte Lawson, Lindsay Bottoms

**Affiliations:** 1 Research Centre for Applied Sport, Physical Activity and Performance, School of Sport & Health Sciences, Faculty of Allied Health and Wellbeing, University of Central Lancashire, Preston PR1 2HE, UK; 2 Gastroenterology Department, Lister Hospital, Stevenage SG1 4AB, UK; 3 School of Life and Medical Sciences, University of Hertfordshire, Hatfield, AL10 9AB, UKl.bottoms@herts.ac.uk (L.B.); 4 Department of Comparative Biomedical Sciences, Royal Veterinary College, London NW1 0TU, UK

**Keywords:** ulcerative colitis, inflammatory bowel disease, Montmorency tart cherry, complimentary medicine, randomized controlled trial

## Abstract

Ulcerative colitis, characterized by its relapsing and remissive nature, negatively affects perception, body image, and overall quality of life. The associated financial burden underscores the need for alternative treatment approaches with fewer side effects, alongside pharmaceutical interventions. Montmorency tart cherries, rich in anthocyanins, have emerged as a potential natural anti-inflammatory agent for ulcerative colitis. This manuscript outlines the study protocol for a randomized placebo-controlled trial investigating the effects of Montmorency tart cherry in individuals with ulcerative colitis. The trial aims to recruit 40 participants with mild to moderate disease activity randomly assign them to either a Montmorency tart cherry or placebo group. The intervention will span 6 weeks, with baseline and 6-week assessments. The primary outcome measure is the Inflammatory Bowel Disease Quality of Life Questionnaire. Secondary outcomes include other health-related questionnaires and biological indices. Statistical analysis will adhere to an intention-to-treat approach using linear mixed effect models. Ethical approval has been obtained from the University of Hertfordshire (cLMS/SF/UH/05240), and the trial has been registered as a clinical trial (NCT05486507). The trial findings will be disseminated through a peer-reviewed publication in a scientific journal.

## 1. Introduction

Ulcerative colitis (UC) is an inflammatory bowel disease that is denoted by acute, non-infectious relapsing and remitting inflammation of the intestinal mucosa [1]. In the United States, there is a prevalence as high as 500 per 100,000 individuals [2], with similar incidence rates noted in the United Kingdom [3] and the prevalence of UC is increasing in many other parts of the developed world [4]. The rising frequency, high morbidity, and fiscal burden of UC are a significant clinical concern. The average direct cost of UC has been estimated to be > USD 3500 per patient, much of which is attributed to medications [5], with one UK drug estimated to cost GBP 400 million per year [6]. Indeed, recent healthcare economic analyses have shown that pharmaceutical agents utilized for UC represent some of the most expensive medications within the US health service [7]. Therefore, UC requires additional financial resources and infrastructure for efficient, long-term disease management [4]. The direct fiscal impact of this condition in the United Kingdom alone exceeds GBP 720 million per annum [8], with prevalence increasing by 0.3% per year in Europe [9].

UC commonly emerges during early adulthood, and its fluctuating and recurring nature can significantly impact patients’ overall health perception, body image, and quality of life [10]. This effect is particularly notable in individuals experiencing active disease, as they tend to report a diminished quality of life compared to those in remission [10]. Patients with UC often exhibit higher levels of psychological distress, anxiety, heightened sensitivity to bodily sensations, and perceive lower levels of social support when compared to healthy individuals [11]. Various factors contribute to a reduced health-related quality of life, including diminished participation in social activities and the impact on relationships with friends, family members, and romantic partners [12]. Furthermore, UC is associated with decreased participation in the labour force, which further amplifies the economic consequences of the condition [13].

The primary objective of treatment for UC is to suppress inflammation [14]. For individuals with mild to moderate disease symptoms, 5-aminosalicylic acid is commonly prescribed [15]. However, the response rates to these agents are typically around 40–50% [16] and can lead to substantial side effects such as hepatitis, pancreatitis, nephritis/nephrotoxicity, and pulmonary dysfunction, and in some cases, they may even worsen the severity of the disease [16]. In cases of moderate to severe UC, corticosteroids and immunosuppressive medicines are often utilized [17]. However, the improvement achieved with this approach is usually protracted and also comes with the risk of significant side effects, including an increased susceptibility to non-Hodgkin’s lymphoma, allergic reactions, hepatic/pancreatic inflammation, infection, tuberculosis, and viral hepatitis [18].

UC is associated with excessive reactive oxygen species levels, generated by activated macrophages and neutrophils within the inflamed intestine [19], as well as imbalances of oxidative stress [20] and diminished capacity of the antioxidant defense system within the intestines [21]. Alternative treatment modalities are increasingly being sought for the management of UC [22]. Recently, more emphasis on dietary therapies have been advocated for UC, as it is thought to alter the composition of gut microbiota [23]. It has been further hypothesized owing to their potential to attenuate inflammation of the gastrointestinal tract, that natural antioxidative and anti-inflammatory agents might be beneficial and offer a treatment approach with fewer side effects compared to pharmaceutical interventions, and recent clinical and animal studies support this notion [14,24]. Anthocyanins form a crucial sub-class of dietary flavonoids and are abundant in dark fruits where they provide the deep colouring in these fruit groups [25]. Importantly, it has been shown that anthocyanins mediate significant antioxidant properties and have been demonstrated to improve and prevent heart disease and certain cancer modalities, in addition to inflammatory pathologies [26], although research concerning their efficacy in UC remains in its infancy.

Animal models show that anthocyanin-rich bilberry ingestion attenuates the severity of disease activity and reduces pro-inflammatory cytokine serum levels [27]. Two subsequent interventions in human participants showed firstly that 6 weeks of bilberry supplementation significantly improved endoscopic disease activity and faecal calprotectin levels [14] and reduced amounts of the pro-inflammatory cytokines IFN-γ and TNF-α from colon biopsies as well as reduced serum levels of TNF-α and MCP-1 [26]. Montmorency tart cherries are considered a rich source of anthocyanins [28], and supplementation has been demonstrated to increase Bacteroides in the gut [29], known to decrease colon inflammation in IBD [30]. Biochemical investigations have shown that the anthocyanin content of Montmorency tart cherries is greater than the majority of dark fruits [28], which has led to the idea that their clinical efficacy may be greater than those observed previously using bilberry supplementation in patients with UC.

### 1.1. Rationale

There are no placebo-controlled, randomized trials examining the efficacy of Montmorency tart cherry in patients with UC. Our research [31] has revealed a strong willingness to take Montmorency tart cherry supplementation in patients with UC, demonstrating a high-level of patient engagement for a randomized clinical trial involving Montmorency tart cherry supplementation. Therefore, further exploration of the health benefits of tart cherries in patients with UC may be of both practical and clinical relevance.

### 1.2. Aims and Objectives

The objective of this trial, is to assess the impact of twice daily Montmorency tart cherry supplementation, on various health indicators in patients with UC, in comparison to the placebo. The primary aim of this randomized trial is to investigate the effects of Montmorency tart cherry supplementation on self-reported quality of life, specifically using the Inflammatory Bowel Disease Quality of Life Questionnaire, in comparison to the placebo group. Secondary objectives involve evaluating whether tart cherry supplementation has any influence on other health-related questionnaires and biological indices.

## 2. Experimental Design

### 2.1. Study Design and Setting

**Figure 1:**
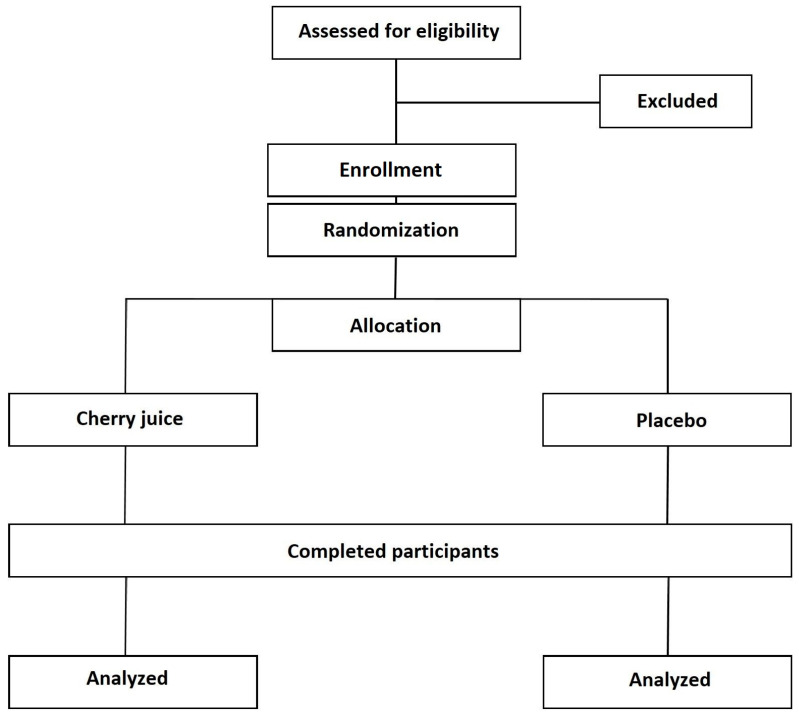
Consort diagram describing the study design.

This study adheres to the latest guidelines for reporting parallel-group randomized trials [32]. The University of Hertfordshire in Hertfordshire, South England, will serve as the location for the trial. The research follows a 6-week parallel design, incorporating randomized allocation with a placebo control (Figure 1). Upon confirming a participant’s suitability and enrollment, a computer program (random assignment software) will individually assign them to one of two groups for the duration of 6 weeks: (1) Montmorency tart cherry supplement or (2) placebo. The primary and secondary outcome variables will be assessed at baseline and after 6 weeks, as described below. Consistent with previous studies on UC management, the primary outcome will be on examining the differences between groups in the Inflammatory Bowel Disease Quality of Life Questionnaire questionnaire between the two groups. Secondary outcomes will be other health-related questionnaires and biological indices.

### 2.2. Inclusion Criteria

To be eligible for inclusion in this study, participants must meet the following criteria: (1) have a confirmed diagnosis of UC for a minimum of 6 months, (2) exhibit mild to moderate disease activity at the time of enrollment, (3) fall within the age range of 18 to 65 years, and (4) have maintained consistent use of medication for a minimum of 3 months. The aforementioned inclusion criteria were collectively adopted based on previous randomized trials in patients with UC [33,34]. Mild–moderate disease activity will be given a Mayo Clinic Score between 3–10, in accordance with the British Society of Gastroenterology consensus guidelines [35].

### 2.3. Exclusion Criteria

Exclusion criteria for this trial include the presence of diabetes, HIV, rheumatoid arthritis, or any other autoimmune disease, as well as hepatitis B and C infections. Additionally, individuals with abscesses, unstable medical conditions that may hinder their ability to complete the study or known food allergies to cherries will also be excluded from participation.

### 2.4. Sample Size

There has yet to be any investigation examining the efficacy of Montmorency tart cherry in patients with UC. Therefore, a pragmatic a priori sample size calculation was undertaken based on a previous trial examining the effects of bilberry supplementation on UC health-related quality of life change scores quantified using the Short Inflammatory Bowel Disease Questionnaire [14]. Considering an expected attrition rate of 20%, this revealed that 20 participants would be necessary in each trial arm, with a total N of 40, to achieve *α* = 5% and *β* = 0.80, enabling an increase in health-related quality of life of 6.5 points to be detected.

### 2.5. Participants and Recruitment

Both males and females of diverse races and ethnicities, who live in Hertfordshire and its surrounding areas, will be recruited. Recruiting materials will be placed via local Crohn’s and Colitis UK (CCUK) network, the CCUK website as well as using social media. We envisage that participant recruitment will commence in September of 2023.

## 3. Procedures

### 3.1. Intervention and Control Groups

After completing their initial data collection, participants will be given Montmorency tart cherry concentrate or a placebo.

#### 3.1.1. Montmorency Tart Cherry

For a period of six weeks, participants assigned to this group will consume 60 mL of Montmorency tart cherry concentrate (Montmorency Tart Cherry Juice Concentrate, King Orchards, Kewadin, MI, USA) each day [36,37,38]. The concentrate will then be diluted with 100 mL of water to make each 30 mL serving into a 130 mL beverage. Each day, 130 mL of the tart cherry supplement will be taken in two equal doses: in the morning and in the evening. A 30 mL dose of Montmorency tart cherry concentrate includes 80 Kcal, 19 g of carbohydrates, of which 15 g are sugars, 1.1 g of protein, and 1 g of fibre, according to the manufacturer. Previous phytochemical analyses have shown that 30 mL of Montmorency tart cherry concentrate contains 9.117 mg/mL of anthocyanins and 6.63 total phenolics/mL (expressed as gallic acid equivalents) [38,39]. However, to quantitatively assess the anthocyanin and phenolic contents of the supplement batch that is utilized in this trial, a phytochemical content analysis of the Montmorency tart cherry concentrate batch will be completed by the manufacturer. Anthocyanin and phenolic contents will be examined using the pH-differential and modified Folin–Ciocalteu colorimetric methods, respectively [40].

#### 3.1.2. Placebo

The placebo will be prepared in accordance with previous trials, that have demonstrated this form of placebo production to be an efficient blinding strategy [36,37]. For a total of six weeks, participants assigned to the placebo group will ingest 60 mL each day [36,37]. The placebo will also be diluted with 100 mL of water and taken twice day in equal amounts, just like the supplement condition. Using a magnetic stirrer (Stuart Scientific, Staffordshire, UK) and stir bar (Fisher Scientific, Waltham, MA, USA), the placebo will be prepared by combining 100% unflavored maltodextrin carbohydrates (MyProtein, Cheshire, UK) with drinking water. Maltodextrin will be combined with water to make 1.0 litre of placebo concentrate, resulting in 20 g of maltodextrin per 30 mL dosing, resembling the carbohydrate/sugar content of the Montmorency tart cherry supplement. In order to replicate the colour of the Montmorency tart cherry concentrate, equal amounts of red and black food colouring will be added. To achieve the desired flavour, cherry flavdrops (1 mL) (MyProtein, Cheshire, UK) will then be added. A 30 mL dose of placebo concentrate includes 0 mg of anthocyanins and contains 100 Kcal, 20 g of carbohydrates, of which 0 g are sugars, as well as 0 g protein and 0 g fibre.

### 3.2. Data Collection

All measurements will be made inside the Physiology laboratory at the University of Hertfordshire and will be carried out in the exact same way on two occasions: at baseline and after six weeks.

#### 3.2.1. Questionnaires

The primary outcome measure for this study will be the Inflammatory Bowel Disease Quality of Life Questionnaire. Secondary questionnaires will include the simple clinical colitis activity index (SCCAI), IBD Fatigue Scale, International Physical Activity Questionnaire—Short Form (IPAQ-SF), and the Hospital Anxiety and Depression Scale. The Inflammatory Bowel Disease Quality of Life Questionnaire is a widely used and validated instrument, specifically designed for patients with IBD [41,42]. It consists of 32 items scored on a 7-point Likert scale, with higher scores indicating better IBD-related quality of life based on a range of 32–224. The questionnaire assesses four dimensions: bowel symptoms, systemic symptoms, emotional well-being, and social functioning. The simple clinical colitis activity index (SCCAI) is a validated, symptom-based index comprising six items. Scores on this index range from 0 to 19, with higher scores indicating more severe symptoms [43]. The IBD Fatigue Scale is an IBD-specific tool that evaluates fatigue in individuals with IBD [44]. It consists of two components: one estimates the level of fatigue using five questions, and the other measures the impact of fatigue on quality of life using 30 questions. Scores are calculated on a 0–4 Likert scale, with a maximum score of 120 and higher scores indicating higher levels of fatigue and greater impact on quality of life. The International Physical Activity Questionnaire—Short Form (IPAQ-SF) is a validated questionnaire consisting of nine items. It assesses physical activity across four intensity levels: vigorous-intensity activity, moderate-intensity activity, walking, and sitting. Energy expenditure is calculated in metabolic equivalents per week [45]. The Hospital Anxiety and Depression Scale is a validated 14-question scale used to assess symptoms of anxiety and depression. It comprises separate sections for depression (7 questions) and anxiety (7 questions), with scores ranging from 0 to 21 for each section. Higher scores indicate greater levels of depression or anxiety [46].

In accordance with previous trials examining the clinical efficacy of Montmorency tart cherries, participants will be required to maintain their habitual patterns throughout the intervention period and also asked to keep a 4-day diet diary prior to the baseline assessment and before the follow-up examination at the end of the 6-week treatment period [36,47,48,49]. This will ensure that there are no differences in dietary patterns between groups and that participants have not made significant changes to their nutritional approach that could influence the study outcomes. Any comorbidities and medications taken by participants will also be recorded.

#### 3.2.2. Biological Samples

To be able to identify biological changes in intestinal inflammation levels, faecal samples for analysis of faecal calprotectin (the gold standard of intestinal inflammation) [50], will be taken from both groups at baseline, and after the 6-week treatment period. Microbiota communities in the gut will also be measured from stool samples to determine changes in the communities. The methods will utilise 16S rRNA gene technology, where sequencing will be completed on Illumina MiSeq for targeted amplicon sequencing and NextSeq for untargeted bacteriophage sequencing. The method will allow the exploration of associations between gut communities, calprotectin, and other metadata. In brief for bacterial community sequencing, raw, paired-end .fastq sequencing files are quality filtered (phred > Q30), merged, trimmed, and clustered in to OTUs (operational taxonomics units) at 97% similarity. Chimeric and non-bacterial reads are removed before taxonomic assignment by alignment to the SILVA or Greengenes databases. Taxonomic classifications are merged at the genus level. Taxonomic abundances are normalized via rarefaction or conversion to proportional abundances. Mean alpha diversity measures, including taxonomic richness and Shannon diversity, are compared between cohorts using generalized linear mixed models. Weighted Bray–Curtis dissimilarity is the measure of beta diversity employed to assess dissimilarities in community structure between cohorts using PERMANOVA. Differential bacterial taxa between cohorts are identified using Maaslin2. Where multiple pairwise comparisons are made, suitable multiple-hypothesis correction is applied.

For untargeted sequencing of the phage fraction on the Illumina NovaSeq. Raw, paired-end .fastq sequences are filtered for quality (phred > Q30), length, (>100 n) and reads aligning to human genomes. Contiguous sequences are assembled before filtering based on read mapping and confirmation of viral origin. Taxonomic assignment at the species level is performed by alignment to relevant viral databases (NCBI, GPD, etc.) Analyses of alpha and beta diversity as well as differential taxa identification are performed as per bacterial community methods. Bacterial and viral communities are normalised via cumulative sum scaling and centered log ratio transformation before MixOmics is used to identify co-abundant features of bacterial and bacteriophage communities associated with cohort membership or intervention group.

#### 3.2.3. Blood Samples

Venous hematological samples will be collected from the antecubital vein (12 mL) directly into uncoated blood collection tubes (Fisher Scientific, Pittsburgh, PA, USA). Blood will be allowed to clot for 30 min before serum separation by centrifugation at 1000× *g*. Serum will be aliquoted and stored at −80 degrees C until assayed. We will use a multiplex approach and develop a bespoke panel (BioLegend Legend Plex) to enable measurement of pro-inflammatory and anti-inflammatory cytokines TNF-a, IL-6, IL-17A, IL-12/IL-23 and anti-inflammatory cytokines IL-10, TGF-beta using a BD FACS CANTO II flow cytometer according to the Legend Plex set up instructions.

### 3.3. Data Management

The Data Protection Act of 2018’s standard guidelines will be followed in the data-gathering and storage processes. Electronic spreadsheets will be used to input data, and Microsoft OneDrive will be used to store them on a secure university server. All information will be handled in confidence and made anonymous for analysis. For the length of the study, hard copies of the data and documents will be stored in a locked filing cabinet. Data will be transmitted to the University of Central Lancashire Research Data Archive (CLOK) after the study is finished, where they will be stored for 7 years. After this time, hard copies will be confidentially disposed of, and electronic data will be erased.

### 3.4. Statistical Analysis

Continuous experimental variables will be presented as mean values along with their corresponding standard deviations. To compare participant characteristics at baseline, as well as compliance levels, experimental anthocyanin intake, experimental energy intake, and experimental sugars (g/day) between the trial arms, between-group linear mixed effects models will be utilized. In these models, the group will be treated as a fixed factor, and random intercepts will be included for participants.

All analyses of the intervention-based data will follow an intention-to-treat approach. To determine the effects of the intervention on all of the outcome measures, differences in the changes from baseline to 6 weeks between the two trial arms will be examined using linear mixed-effects models with a group modelled as a fixed factor and random intercepts by participants adopted. For linear mixed models, the mean difference between groups in changes from baseline to 6 weeks (*b*) and 95% confidence intervals of the difference will be presented. Effect sizes were calculated for the changes from baseline to 6 weeks between the two groups, using Cohen’s *d*, in accordance with McGough & Faraone [51]. Cohen’s d values will be interpreted as 0.2 = small, 0.5 = medium, and 0.8 = large [52]. The efficacy of blinding will be assessed using a one-way chi-square (*Χ*^2^) goodness-of-fit test. Chi-square analyses will be calculated using Monte-Carlo simulation to determine probability values. All statistical analyses will be performed using SPSS v28 (IBM Inc., SPSS, Chicago, IL, USA), and statistical significance will be considered at a *p* ≤ 0.05 level.

### 3.5. Ethical Approval and Registration

This study has been granted ethical approval by the University of Hertfordshire Health, Science, Engineering and Technology Ethics Committee (cLMS/SF/UH/05240), and has formally been registered as a trial (NCT05486507). When the data have been evaluated, participants who request to see a summary of the study results will be given that information. Publication in a peer-reviewed journal and presentation at both national and global scientific conferences will be the primary means of disseminating the study findings from this trial.

### 3.6. Dissemination

The results and conclusions of this study will be communicated to a broad range of local, national, and international audiences as part of its dissemination strategy. We will include someone with UC on the trial steering group and include them within steering meetings. On completion of the trial, we will disseminate the results through local inflammatory bowel disease support groups. To best facilitate user distribution, we will coordinate with our patient representatives and regional authorities on patient and public involvement. We intend to create a specialized information article that will be distributed to the proper organizations. We will produce peer reviewed publications and present at an international conference.

### 3.7. Safety Reporting

The Sponsor’s Adverse Event Reporting Procedures shall be followed for reporting adverse events. The clinical co-investigator will be in charge of assessing the cause and severity of adverse events, as well as ensuring that appropriate action is done. Data on adverse events will be gathered from the start of any study-related procedure (i.e., upon acquisition of written consent). The participant’s final trial contact will mark the end of the adverse event reporting period.

Throughout the study, we will closely monitor and document both significant and non-serious adverse effects that may be associated with participation in the research or lead to withdrawal from the or study. Serious adverse events encompass any unfavorable medical event. In the event of any necessary modifications to the experimental protocol, we will promptly inform the study ethics committee for reevaluation and approval, and the trial registry will be updated accordingly. During this period, data collection will be temporarily halted. Non-serious incidents, on the other hand, encompass medical occurrences that do not meet the criteria for serious adverse events.

## 4. Expected Results

The proposed placebo randomized controlled trial aims to investigate the effects of Montmorency tart cherry supplementation in individuals with UC. The study seeks to determine the effectiveness of a 6-week intervention involving twice-daily Montmorency tart cherry supplementation on various aspects of the disease. The primary objective of this trial is to assess the impact of the intervention on Inflammatory Bowel Disease Quality of Life, evaluating the overall well-being and quality of life of patients with UC. Additionally, the study will utilize other health-related questionnaires to gather information on different aspects of health. Furthermore, the trial will analyse peripheral blood biomarkers to evaluate the inflammatory response in the body. This will provide insights into whether Montmorency tart cherry supplementation has an effect on reducing UC-related inflammation. Another important aspect of the investigation will be the analysis of the fecal microbiome community. The study will explore whether Montmorency tart cherry supplementation can influence the stability and composition of the gut microbiome, which has been associated with the development and progression of UC.

The predicted outcomes of the study suggest that Montmorency tart cherry supplementation will lead to significant improvements in self-reported quality of life compared to the placebo. Additionally, the researchers anticipate positive changes in health-related questionnaires, peripheral blood biomarkers indicating reduced inflammation, and modulation of the gut microbiome stability compared to the placebo group. Should the findings of this randomized controlled trial support these predictions, they would provide valuable and clinically significant information. Given the debilitating nature of UC, the associated healthcare costs, and its negative impact on quality of life and psychological well-being, the results could have practical implications for improving the management and treatment of UC using Montmorency tart cherry supplementation.

## Data Availability

This is a protocol therefore there is no current data available to share.
